# A genomic approach to study down syndrome and cancer inverse comorbidity: untangling the chromosome 21

**DOI:** 10.3389/fphys.2015.00010

**Published:** 2015-02-04

**Authors:** Jaume Forés-Martos, Raimundo Cervera-Vidal, Enrique Chirivella, Alberto Ramos-Jarero, Joan Climent

**Affiliations:** Genomics and Systems Biology (InGSB) Lab, Oncology and Hematology Department, Biomedical Research Institute INCLIVAValencia, Spain

**Keywords:** down syndrome, cancer genomics, Chr. 21p11, RCAN1, BTG3, inverse comorbidity

## Abstract

Down syndrome (DS), one of the most common birth defects and the most widespread genetic cause of intellectual disabilities, is caused by extra genetic material on chromosome 21 (HSA21). The increased genomic dosage of trisomy 21 is thought to be responsible for the distinct DS phenotypes, including an increased risk of developing some types of childhood leukemia and germ cell tumors. Patients with DS, however, have a strikingly lower incidence of many other solid tumors. We hypothesized that the third copy of genes located in HSA21 may have an important role on the protective effect that DS patients show against most types of solid tumors. Focusing on Copy Number Variation (CNV) array data, we have generated frequencies of deleted regions in HSA21 in four different tumor types from which DS patients have been reported to be protected. We describe three different regions of deletion pointing to a set of candidate genes that could explain the inverse comorbidity phenomenon between DS and solid tumors. In particular we found RCAN1 gene in Wilms tumors and a miRNA cluster containing miR-99A, miR-125B2 and miR-LET7C in lung, breast, and melanoma tumors as the main candidates for explaining the inverse comorbidity observed between solid tumors and DS.

## Introduction

Down syndrome (DS) was first described and named by the British physician John Langdon Down about 150 years ago. However, Professor Jerome Lejeune discovered a century later that the cause of this disorder was the presence of an extra chromosome 21 (HSA21) (Mégarbané et al., 2009). Today, DS is the most widespread cause of genetic intellectual disability, with an approximated prevalence of between 1 in 1000 and 1 in 1100 live births worldwide, and roughly 3000–5000 new cases per year (www.who.int). The Down phenotype is caused by a complete or partial trisomy (TS21) of human chromosome 21 (HSA21) acquired essentially by meiotic non-disjunction events during gametogenesis. Besides the cognitive impairment typical of DS patients, a variable set of associated conditions, such as congenital heart disease, vision problems, hearing loss, or decreased immune system activity, has been linked to the disease (Roizen and Patterson, 2003). In the past two decades, substantial advances in exploring the human genome, together with the exponential growth of bioinformatics, have permitted a better understanding of the functional links between the extra copy of HSA21 and the variations in the different phenotypes of DS individuals (Letourneau and Antonarakis, 2012; Letourneau et al., 2014). The sequencing of HSA21 and the experimental research done with models of DS have allowed the scientific community to connect specific genomic regions and sets of genes to different clinical conditions and syndrome phenotypes. Initially, a total of 225 genes were identified (Hattori et al., 2000), and 2 years later the gene content was updated to 329 genes (Kapranov et al., 2002). Nowadays, based on data provided by large-scale studies, the HSA21 gene number is estimated to be around 534 from a total of 2176 gene transcripts (Scarpato et al., 2014).

New epidemiological insights attempting to define direct and inverse comorbidities between complex disorders have started to reveal the complicated connections that DS and cancer may share. This epidemiological evidence points to both a higher- and a lower-than-expected probability of developing some tumors in patients with DS (Ibáñez et al., 2010; Tabarés-Seisdedos et al., 2011; Tabarés-Seisdedos and Rubenstein, 2013). Catalá-López et al. (2014) conducted a meta-analysis based on published epidemiological studies reporting comorbidities between cancer and CNS disorders, gathering data for 17,090 DS patients. This study showed an increased risk for developing leukemia and testicular cancer in DS patients. Other studies in different ethnographic populations have evidenced a lower incidence of most tumors in patients with DS (Hasle et al., 2000; Boker et al., 2001; Nižetić and Groet, 2012). In particular, breast cancer incidence shows a considerable decrease in DS patients compared with age-matched euploid individuals.

Interestingly, the observed protection against many solid tumors in DS patients has opened a window of opportunity in the search for tumor suppressor genes located on chromosome 21 that would amplify their effect in a dosage-dependent way. Over the last few years, and in parallel to the above, a great amount of research has focused on the associations between genomic alterations, like somatic copy number variations (CNVs), and cancer (Rubio-Moscardo et al., 2005; Climent et al., 2007; Jönsson et al., 2012; Hieronymus et al., 2014). The result of such analyses is a noteworthy amount of open access microarray data available at public repositories such as NCBI Gene Expression Omnibus (GEO, at http://www.ncbi.nlm.nih.gov/geo/), or EBI (ArrayExpress http://www.ebi.ac.uk/arrayexpress/).

In the present perspective, we first approached DS as a genetic disorder caused by the extra copy of genomic DNA on chromosome 21. Our aim was to collect previously published CNV array data from cancers for which there exists an observed decreased incidence in DS as compared to that for non-DS patients. We then screened for the most common structural alterations present in chromosome 21. This might lead to identifying regions containing genes potentially responsible for the protection against solid tumor development in DS individuals. Our underlying hypothesis was that copy gains of tumor suppressor genes in chromosome 21 of DS patients would be responsible for the protection phenomenon observed between DS and most types of solid tumors. Therefore, we expected to find those genes in the maximum frequency deletion regions of tumor samples coming from non-DS patients. To our knowledge, our work presents the first attempt at defining tumor suppressor candidate genes using this approach. We selected four tumor types described as low incidence tumors in DS by epidemiological studies: breast cancer (359 samples), lung cancer (78 samples), melanoma (34 samples) and Wilms tumor (18 samples). Breast and lung cancer showed a standardized incidence ratio (SIR) of 0.4 and 0.24, respectively, between DS and age-matched euploid population cohorts (Hasle et al., 2000; Patja et al., 2006). The SIR value for the skin cancer group, in which melanoma was included, was 0.25. Kidney cancer, including Wilms tumor, showed a SIR value of 0.84 (Hasle et al., 2000; Patja et al., 2006), and no cases of DS were found in a study that included 5854 Wilms tumor patients (Olson et al., 1995).

## Materials and methods

### Array CGH analysis

We analyzed data from multiple platforms to generate both the disease-specific frequencies of amplification and deletion, as well as the frequency summaries for four tumor types described as low incidence tumors in DS by epidemiological studies based on previously published data (Table 1). Raw Array CGH data files were downloaded from Gene Expression Omnibus, and normalization and conversion to log2 values of intensity were performed. Probe annotations from assemblies older than hg19 were remapped to hg19 using Lift Genome Annotation tool, software (http://genome.ucsc.edu/cgi-bin/hgLiftOver) which converts coordinates and genome annotation files between assemblies. To define amplification and deletion regions, we used the R-package CGHcall, which applies the Circular Binary Segmentation (CBS) algorithm (Hsu et al., 2011) it seeks out gain and loss segments by recursively dividing the genome until it identifies segments that have probe distributions different from their neighbors. This was followed by a CGHcall algorithm producing an objective and effective classification of the segmented data into copy number states. Once we had computed platform-specific data for amplification and deletion, we transformed them into multiplatform-comparable data. To do so, we defined 5000 anchor positions along chromosome 21, and performed an estimation of the amplification and deletion value for each of these anchors in every sample for every platform using custom R code (see Supplementary data). Once we had the amplification and deletion values for the anchors, we calculated the frequencies of amplification and deletion for the set of all diseases.

**Table 1:** **Datasets used for the copy number (CN), gene expression (Exp) and microRNA expression (miR) downloaded from Gene Expression Omnibus (GEO)**.

**Tumor type**	**GEO**	**Platform**	**Data**	**References**
Breast	GSE22133	SWEGENE_BAC_33K_Full	CN	Jönsson et al., 2012
Melanoma	GSE45354	Agilent-021924 SurePrint G3 Human CGH Microarray 8 × 60 K	CN	
Lung	GSE29065	SWEGENE_BAC_32K_Full	CN	Staaf et al., 2013
Wilms	GSE28397	Custom Agilent 2 × 105 K Human Genomic microarray	CN	
Breast	GSE22820	Agilent-014850 Whole Human Genome Microarray 4x44K G4112F	Exp	Liu et al., 2011
Melanoma	GSE46517	Affy metrix U133A microarray chip	Exp	Kabbarah et al., 2010
Lung	GSE19804	Affy metrix Human Genome U133 Plus 2.0 Array	Exp	Lu et al., 2010
Wilms	GSE4530	Homo sapiens 4.8K 02-01 amplified cDN A	Exp	
Breast	GSE48088	[miRNA-2_0] Affy metrix Multispecies miRNA-2_0 Array	miR	Peña-Chilet et al., 2014
Melanoma	GSE35579	CRUK/Melton lab-Human melanoma-71 -v2 -microRN A expression	miR	Xu et al., 2012
Lung	GSE51855	Agilent-015508 Human miRNA Microarray	miR	Arima et al., 2014
Wilms	GSE38419	Febit Homo Sapiens miRBase 13.0	miR	Schmitt et al., 2012

### Expression array analysis

We downloaded raw data from GEO, selecting the datasets describing gene expression from both tumor samples, as well as from normal tissue, in order to perform differential expression analysis (see Table 1 below). Data and raw signal intensities were checked for quality, and a quantile normalization was done. A multiple *t*-test was performed to search for differentially expressed genes located on chromosome 21, and the *p*-values were corrected for FDR.

### miRNA expression array analysis

For the miRNA differential expression analysis, we retrieved raw data from studies that included tumor samples and healthy control tissues. We applied quantile normalization and carried out a differential expression analysis using the SAM method implemented in the R package *samr*.

## Results and discussion

**Figure 1:**
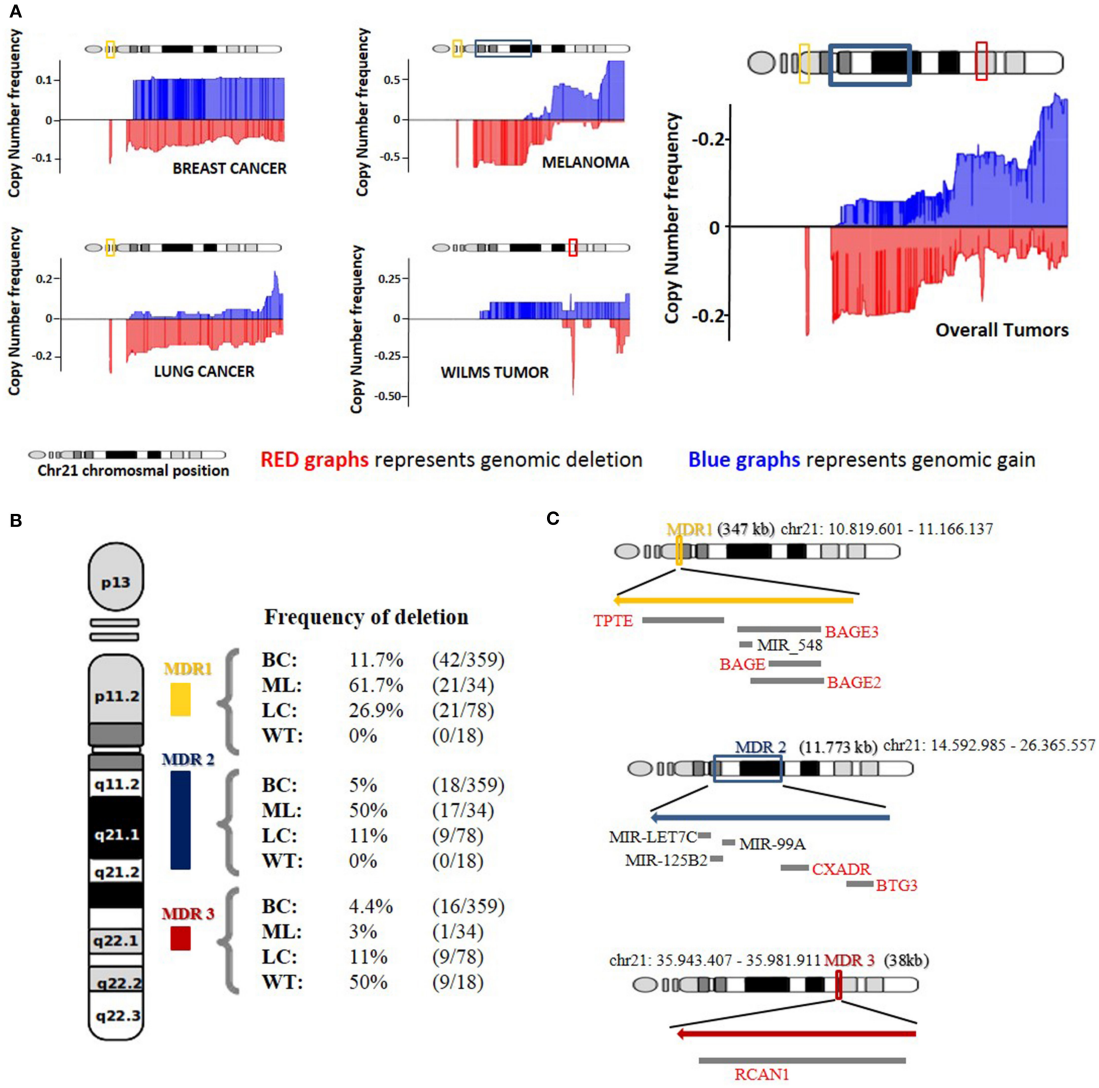
**Copy number analysis of Chromosome 21. (A)** Representation of genomic gain (blue) and deletion (red) frequencies on four different tumor types and median average of all tumors together. **(B)** Deletion frequency for the three major deleted regions (MDR) in breast cancer (BC), melanoma (ML), lung tumors (LT), and Wilms Tumor (WT) on HSA21. **(C)** Representation of the three MDR based on genomic localization and size with those genes and miRNAs that showed downregulation in the gene expression correlation analysis.

Direct comorbidities between DS and cancer have been the subject of several studies that have shed light onto the primary molecular causes of these associations (Catalá-López et al., 2014; Letourneau et al., 2014). Nevertheless, inverse comorbidities are mainly sketched thanks to epidemiological research, and lack molecular explanations (Tabarés-Seisdedos et al., 2011). To better understand how DS patients could be protected from some types of solid tumors, we have analyzed the copy number profiles of chromosome 21 from four cancer types observed with a lower-than-expected incidence in DS patients by epidemiological data (Nižetić and Groet, 2012; Catalá-López et al., 2014). We generated a mean amplification and deletion profile for the four cancer types and observed three principal maximum deletion regions in chromosome 21. We selected as candidate genes those present in the maximum frequency of deletion regions (MDR) that were further found downregulated in at least 2 of the expression datasets analyzed (Figure 1).

Maximum frequency deletion region 1 (MDR1) is observed at cytoband 21p11.2 and spans from position 10,704,090–11,166,137. Strikingly, this region is the maximum deletion region for 3 of the 4 cancer types included in the analysis. Lung and breast cancers and melanoma show deletion frequencies of 26.9, 11.7, and 61.7%, respectively. Wilms tumor does not present deletion in this region. MDR1 contains the genes TPTE and a cluster of different isoforms of the BAGE gene. The BAGE gene has been reported to be a tumor rejection antigen recognized by cytotoxic T cells (Nagel et al., 2003). An impaired expression of the BAGE gene, due to CNVs, could lead to a reduction in immune system targeting and recognition of cancerous cells capacity. The TPTE gene has been described as a transmembrane tyrosine phosphatase related to the PTEN gene and may play a role in signal transduction pathways. PTEN is a validated tumor suppressor gene with phosphatase activity that inhibits the PI3K pathway (Davidson et al., 2010). The actual TPTE *in vivo* function is still unclear, but a hypothetical role in PI3K pathway inhibition could explain how an overexpression of this gene in cells carrying an extra-copy of chromosome 21 could protect from cancer cell proliferation and why MDR1 is the most common deleted region in the three cancer types previously described.

Data regarding the CNVs for BAGE and TPTE agree with the results of the differential expression analysis carried out for breast and lung cancer, where both genes are downregulated. The reduction of the genetic product of those genes could confer advantages in the tumor proliferation process. Moreover, MDR1 contains a micro RNA, miR_548, which recently has been reported to play a possible role in tumor suppression through the control of gene FHIT action in human cancer (Hu et al., 2014). We were not able to detect a downregulation of miR_548 in any of the miRNA expression datasets.

Maximum frequency deletion region 2 (MDR2) is a wide deletion region, mainly found in melanoma, spanning from position 14,592,985 to position 26,365,557 and covering cytobands 21q11.2, 21q21.1 and a segment of 21q21.2. It shows a deletion frequency of 50%, and also appears deleted in breast and lung cancer, displaying a deletion frequency of 5 and 11%, respectively (Figure 1B). MDR2 includes 26 coding genes and 3 miRNAs. Given the high number of genes contained in this region, we focused only on those 2 genes that were also downregulated in at least two of the associated expression datasets.

The BTG3 gene is downregulated both in melanoma and breast cancer, while CXADR is downregulated in lung cancer and melanoma. BTG3 has previously been reported to be downregulated in a wide variety of breast cancer cell lines (Yu et al., 2008) and belongs to a protein family characterized by its antiproliferative properties. Moreover, the low expression of BTG3 has previously been related to the progression of several kinds of cancer, such as adenocarcinoma, oral squamous cell cancer, non-small cell lung cancer, prostate cancer, and hepatocellular carcinoma (Chen et al., 2013; Lv et al., 2013). Additionally, the downregulation of BTG3 is a feature of breast cancer patient samples in the study from Liu et al. (2011). Furthermore, data from the expression analysis suggest that BTG3 downregulation may play an important role in melanoma progression, and to our knowledge, this is a result not previously described in the literature. The molecular function reported for BTG3, along with the large number of solid tumors where its downregulation is observed, indicates that BTG3 may be a good candidate for explaining inverse comorbidity events between DS and cancer.

MDR2 also contains a microRNA cluster containing miR-LET7C,miR-152B2, and miR-99A (Figure 1C). It has been shown that the downregulation of miR-LET7C in prostate cancer is followed by an increase in the androgen receptor (AR) expression levels leading to an increased proliferation of the tumor cells. Additionally, the overexpression of miR-LET7C leads to an impairment of AR molecules entailing a reduction in the proliferative capacity of the cancerous cells (Nadiminty et al., 2012). Moreover, low levels of miR-LET7C have been reported in lung tumors associated with high levels of Ras protein, which activate cell proliferation via the MAPK pathway (Johnson et al., 2005). miR-125B2 has been reported to be downregulated in several tumor types, such as breast, prostate, ovarian and neuroblastoma, among others (Iorio et al., 2005; Ozen et al., 2008), indicating a putative tumor suppressor function. Finally, the downregulation of miR-99A has been linked to increased proliferative capacities in different cancer types (Sun et al., 2011; Chen et al., 2012; Xin et al., 2013). We found miR-99A downregulated in the lung cancer, breast cancer and melanoma datasets, miR-125B2 in breast cancer and melanoma datasets and miR-LET7C in the melanoma and Wilms tumor datasets. Altogether, this could indicate that the extra copy of chromosome 21 in DS patients could lead to an increased expression of the microRNA cluster, leading to a synergic effect over cell proliferation. These miRNAs could be good candidates for explaining the inverse comorbidity observed between solid tumors and DS.

To delve deeper into this notion, we used the known gene targets of the 3 miRNAs to perform a REACTOME (Croft et al., 2014) pathway enrichment analysis. The results of the analysis showed that the set of validated target genes for the three miRNAs is enriched in pathways related to cell proliferation or apoptosis inhibition, like PI3K/AKT activation (REACT_12464), signaling by ERBB2 (REACT_115755) and signaling by NOTCH, Interferon alpha/beta and IFG1R (REACT_299, REACT_25162 and REACT_150210, respectively). These enrichments are consistent with their theoretical role as candidate genes for the inverse comorbidity phenomenon observed between DS and solid tumors.

The last maximum frequency deletion region we describe in our study, MDR3, is a very small region on Wilm's tumors and is deleted in 50% of the samples analyzed. It covers a narrow segment between positions 35,943,407 and 35,981,911, and it includes only one gene, RCAN1 (Figure 1C). The protein encoded by this gene, previously known as DSCR1 (DS critical region gene 1), interacts with calcineurin-inhibiting calcineurin-dependent signaling pathways (Carme Mulero et al., 2010). RCAN1 is located in the minimal candidate region of the DS phenotype, an area of approximately 3 Mb at chromosomal region 21q22 (OMIM 602917), and it is overexpressed in the DS patient's brain. Its overexpression is linked to the formation of neurofibrillary tangles in Alzheimer's disease patients and with the facilitation of neural apoptosis in the DS phenotype (Sun et al., 2014). Comparative sequence analyses of HSA21 and the mouse genome models of DS resulted in the discovery of DSRC1 (RCAN1) as a potential candidate for being a tumor suppressor gene in both lung tumor, as well as melanoma cells, through the suppression of tumor angiogenesis (Minami et al., 2014; Shin et al., 2014). These findings, combined with the observed frequency of deletion shown in Wilms tumor, to our knowledge has not been previously reported. Moreover, the general downregulation trend present in three of the four tumor types analyzed here, lung cancer, breast cancer and Wilms tumor, point to RCAN1 as the best supported link between the Down phenotype and protection from cancer.

In conclusion, although we admit the possible existence of bias as a result of the small number of samples, our genomic approach demonstrates that the analysis of Chromosome 21-deleted regions from specific tumors may allow the prediction of candidate genes to explain why individuals with DS have a reduced risk for developing these tumors. Further functional studies, however, are needed to prove the potential tumor suppressor properties of these candidate genes and miRNA.

### Conflict of interest statement

The authors declare that the research was conducted in the absence of any commercial or financial relationships that could be construed as a potential conflict of interest.
