# Supplementary material for: A genomic approach to study down syndrome and cancer inverse comorbidity: untangling the chromosome 21
Source: Front Physiol. 2015 Feb 4;6:10. doi: 10.3389/fphys.2015.00010 (PMC4316712; doi:10.3389/fphys.2015.00010)
Supplement: Supplementary file 1 [file Table1.DOCX]

#CUSTOM R CODE FOR CGH ANALYSIS

#Chromosome lengths from each assembly.

.getChromosomeLengths <- function(build) {

build <- as.integer(gsub('[^0-9]', '', build))

if (build == 34 || build == 16) {

chromosome.lengths <- c(246127941, 243615958, 199344050, 191731959, 181034922, 170914576, 158545518, 146308819, 136372045, 135037215, 134482954, 132078379, 113042980, 105311216, 100256656, 90041932, 81860266, 76115139, 63811651, 63741868, 46976097, 49396972, 153692391, 50286555)

} else if (build == 35 || build == 17) {

chromosome.lengths <- c(245522847, 243018229, 199505740, 191411218, 180857866, 170975699, 158628139, 146274826, 138429268, 135413628, 134452384, 132449811, 114142980, 106368585, 100338915, 88827254, 78774742, 76117153, 63811651, 62435964, 46944323, 49554710, 154824264, 57701691)

} else if (build == 36 || build == 18) {

chromosome.lengths <- c(247249719, 242951149, 199501827, 191273063, 180857866, 170899992, 158821424, 146274826, 140273252, 135374737, 134452384, 132349534, 114142980, 106368585, 100338915, 88827254, 78774742, 76117153, 63811651, 62435964, 46944323, 49691432, 154913754, 57772954)

} else {

chromosome.lengths <- c(249250621, 243199373, 198022430, 191154276, 180915260, 171115067, 159138663, 146364022, 141213431, 135534747, 135006516, 133851895, 115169878, 107349540, 102531392, 90354753, 81195210, 78077248, 59128983, 63025520, 48129895, 51304566, 155270560, 59373566)

}

names(chromosome.lengths) <- 1:24

return(chromosome.lengths)

}

#Gene content Hash table generator from UCSC.

hashtable <- function(hg = "hg19"){

library(RMySQL)

#dbClearResult(rs)

con <- dbConnect(MySQL(), user="genome",dbname=hg,host="genome-mysql.cse.ucsc.edu")

rs <- dbSendQuery(con, "SELECT kgID, geneSymbol, mRNA, spID FROM kgXref")

data <- fetch(rs, n = -1)

rs2 <- dbSendQuery(con, "SELECT * FROM knownToLocusLink;")

data2 <- fetch(rs2, n = -1)

rsmap <- dbSendQuery(con, "SELECT name, chrom, txStart, txEnd, strand FROM refGene;")

datamap <- fetch(rsmap, n = -1)

names(datamap)[1] <- "mRNA"

names(data)[1] <- paste("name")

df.parcial <- merge(data,data2,by="name")

df.total <- merge(df.parcial,datamap,by="mRNA")

df.total.filt <- df.total[df.total$chrom %in% c("chr1","chr2","chr3","chr4","chr5","chr6","chr7","chr8","chr9","chr10","chr11","chr12","chr13","chr14","chr15","chr16","chr17","chr18","chr19","chr20","chr21","chr22","chrX","chrY"),]

hashchrom <- generadorHashTraductor()

for ( i in 1:nrow(df.total.filt)){

print(i)

df.total.filt[i,"chrom"] <- get(df.total.filt[i,"chrom"],hashchrom)

}

return(df.total.filt)

}

#Get genes from selected region.

sacarGenes <- function(inicio,fin,chr,tabla){

cromosomas <- tabla$chrom

cromo.bool <- cromosomas == chr

tabla.filt <- tabla[cromo.bool,]

tabla.filt.txStart <- tabla.filt$txStart

start.bool <- tabla.filt.txStart >= inicio

tabla.filt.txEnd <- tabla.filt$txEnd

end.bool <- tabla.filt.txEnd <= fin

tabla.conjunta <- start.bool & end.bool

filtrado <- tabla.filt[tabla.conjunta,]

Entrez <- filtrado$value

return (Entrez)

}

#Amplication and deletion regions frequency generator.

recuentoAmpAndDel <- function(df,num){

vectorAmp <- c()

vectorDel <- c()

#print(vectorDel)

for ( i in 1:nrow(df) ){

print(i)

valAuxAmp <- 0

valAuxDel <- 0

for ( j in 4:ncol(df)){

if (df[i,j] > 0){

valAuxAmp <- valAuxAmp + 1

}

if (df[i,j] < 0){

valAuxDel <- valAuxDel + 1

}

}

vectorAmp <- c(vectorAmp,valAuxAmp)

vectorDel <- c(vectorDel,valAuxDel)

}

vectorAmpDef <- c()

vectorDelDef <- c()

for ( i in 1:length(vectorAmp)){

print(i)

if ( vectorAmp[i] == 0 ){

vectorAmpDef <- c(vectorAmpDef,0)

}else{

vectorAmpDef <- c(vectorAmpDef,vectorAmp[i]/num)

}

if ( vectorDel[i] == 0 ){

vectorDelDef <- c(vectorDelDef,0)

}else{

vectorDelDef <- c(vectorDelDef,vectorDel[i]/num)

}

}

dfa <- df[,1:3]

dfinal <- cbind(dfa,vectorAmpDef)

dfinal <- cbind(dfinal,vectorDelDef)

colnames(dfinal) <- c("chr", "ini", "fin", "amp", "del")

return(dfinal)

}

#Mean spacing between probes

CalcularEspaciadoPromedio <- function(Object){

vectorDistancias <- c()

for ( i in 2:nrow(Object)-1){

vectorDistancias <- c(vectorDistancias,Object[i+1,"ini"] - Object[i,"fin"])

}

media <- sum(vectorDistancias)/length(vectorDistancias)

return(media)

}

#Left and right clone selector

BuscarClones <- function(objeto,posicion){

vector.iz.bool <- objeto[,"fin"] < posicion

vector.der.bool <- objeto[,"ini"] > posicion

#print (vector.iz.bool)

pos.ant <- 'NA'

pos.post <- 'NA'

for ( i in 1:length(vector.iz.bool)){

if ( vector.iz.bool[i] == FALSE ){

pos.ant <- i - 1

break

}

}

for ( i in 1:length(vector.der.bool)){

if ( vector.der.bool[i] == TRUE){

pos.post <- i

break

}

}

antpos <- c(pos.ant,pos.post)

print (antpos)

return(antpos)

}

#Anchor generator

CalcularAnclas <- function(lonchr,numeroseg){

espaciado <- (lonchr / 5000)

anclas <- seq(from = 1, to = lonchr, by = espaciado)

return ( anclas )

}

AnclaDentroAnclaFuera <- function(ancla,objeto){

a <- objeto[,"ini"] <= ancla

b <- objeto[,"fin"] >= ancla

c <- a & b

suma <- sum(c)

if (suma > 0){

return (TRUE)

}else{

return (FALSE)

}

}

#Anchor not inside clone frequency value generator

BuscarClones2 <- function(objeto,ancla){

espaciado.promedio <- CalcularEspaciadoPromedio(objeto)

pos.iz <- NA

pos.der <- NA

clon.iz.bool <- objeto[,"fin"] < ancla

suma.iz <- sum(clon.iz.bool)

if ( suma.iz > 0 ){

which.iz <- which(clon.iz.bool == TRUE)

pos.iz <- which.iz[[length(which.iz)]]

}

clon.der.bool <- objeto[,"ini"] > ancla

suma.der <- sum(clon.der.bool)

if (suma.der > 0){

pos.der <- which(clon.der.bool == TRUE)[[1]]

}

#print (pos.iz)

if ( !is.na(pos.iz)){

dif.iz <- ancla - objeto[pos.iz,"fin"]

#print (dif.iz)

if ( dif.iz > espaciado.promedio){

pos.iz <- NA

}

}

if (!is.na(pos.der)){

dif.der <- objeto[pos.der,"ini"] - ancla

#print (dif.der)

if (dif.der > espaciado.promedio){

pos.der <- NA

}

}

return (c(pos.iz,pos.der))

}

#Anchor inside clone frequency generator.

valor.dentro.clon <- function(objeto,ancla,mode){

a <- objeto[,"ini"] < ancla

b <- objeto[,"fin"] > ancla

c <- a & b

freq <- objeto[c,mode][[1]]

}

#Anchor frequency generator.

CalcularFrecuenciasAnclas2 <- function(objeto,longchr,numeroAnclas,mode){

frec.anclas <- c()

anclas <- CalcularAnclas(longchr,numeroAnclas)

for ( i in 1:length(anclas)){

print (i)

#print (frec.anclas)

if (AnclaDentroAnclaFuera(anclas[i],objeto)){

frec.anclas <- c(frec.anclas,valor.dentro.clon(objeto,anclas[i],mode))

}else{

clones <- BuscarClones2(objeto,anclas[i])

if ( is.na(clones[1]) & ! is.na(clones[2]) ){

frec.anclas <- c(frec.anclas,objeto[clones[2],mode])

}

if ( ! is.na(clones[1]) & is.na(clones[2])){

frec.anclas <- c(frec.anclas,objeto[clones[1],mode])

}

if ( is.na(clones[1]) & is.na(clones[2])){

frec.anclas <- c(frec.anclas,0)

}

if ( ! is.na(clones[1]) & ! is.na(clones[2])){

if (objeto[clones[1],mode] == objeto[clones[2],mode] ){

frec.anclas <- c(frec.anclas,objeto[clones[1],mode])

}else{

frec.anclas <- c(frec.anclas,(objeto[clones[1],mode] + objeto[clones[2],mode])/2)

}

}

}

}

return(frec.anclas)

}

#From Entrez ID to Gene Symbol ID.

fromEntrezToSymbol <- function(listaEntrez,tablahg.ordenada){

listaSimbolos <- c()

for ( i in 1:length(listaEntrez)){

tabla.bool <- tablahg.ordenada[,"value"] == listaEntrez[i]

tabla.filt <- tablahg.ordenada[tabla.bool,]

listaSimbolos <- c(listaSimbolos,tabla.filt[1,"geneSymbol"])

}

listaSimbolos <- unique(listaSimbolos)

return(listaSimbolos)

}

#Chromosome banding.

generarBandeo <- function(chr){

library(RMySQL)

con <- dbConnect(MySQL(), user="genome",dbname="hg19",host="genome-mysql.cse.ucsc.edu")

rs <- dbSendQuery(con, "SELECT chrom, chromStart, chromEnd, name, gieStain FROM cytoBand WHERE chrom like 'chr21';")

data <- fetch(rs, n = -1)

data

return(data)

}

imagenBandeo21 <- function(width = 0.005, alt = 0.20){

rect <- data.frame (xmin=0, xmax=2800000, ymin = -(width) + alt, ymax = width + alt)

c <- p + geom_rect(data=rect, aes(xmin=xmin, xmax=xmax, ymin=ymin, ymax=ymax), color="grey20", alpha=0.2, inherit.aes = FALSE)

rect <- data.frame (xmin=2800000, xmax=6800000, ymin = -(width) + alt, ymax = width + alt)

c <- c + geom_rect(data=rect, aes(xmin=xmin, xmax=xmax, ymin=ymin, ymax=ymax), color="grey40", alpha=0.5, inherit.aes = FALSE)

rect <- data.frame (xmin=6800000, xmax=10900000, ymin = -(width) + alt, ymax = width + alt)

c <- c + geom_rect(data=rect, aes(xmin=xmin, xmax=xmax, ymin=ymin, ymax=ymax), color="grey20", alpha=0.2, inherit.aes = FALSE)

rect <- data.frame (xmin=10900000, xmax=13200000, ymin = -(width/2) + alt , ymax = width/2 + alt)

c <- c + geom_rect(data=rect, aes(xmin=xmin, xmax=xmax, ymin=ymin, ymax=ymax), color="grey40", alpha=0.5, inherit.aes = FALSE)

rect <- data.frame (xmin=13200000, xmax=14300000, ymin = -(width/2) + alt, ymax = width/2 + alt)

c <- c + geom_rect(data=rect, aes(xmin=xmin, xmax=xmax, ymin=ymin, ymax=ymax), color="grey20", alpha=0.5, inherit.aes = FALSE)

rect <- data.frame (xmin=14300000, xmax=16400000, ymin = -(width) + alt, ymax = width + alt)

c <- c + geom_rect(data=rect, aes(xmin=xmin, xmax=xmax, ymin=ymin, ymax=ymax), color="grey20", alpha=0, inherit.aes = FALSE)

rect <- data.frame (xmin=16400000, xmax=24000000, ymin = -(width) + alt, ymax = width + alt)

c <- c + geom_rect(data=rect, aes(xmin=xmin, xmax=xmax, ymin=ymin, ymax=ymax), color="grey20", alpha=0.8, inherit.aes = FALSE)

rect <- data.frame (xmin=24000000, xmax=26800000, ymin = -(width) + alt, ymax = width + alt)

c <- c + geom_rect(data=rect, aes(xmin=xmin, xmax=xmax, ymin=ymin, ymax=ymax), color="grey20", alpha=0, inherit.aes = FALSE)

rect <- data.frame (xmin=26800000, xmax=31500000,ymin = -(width) + alt, ymax = width + alt)

c <- c + geom_rect(data=rect, aes(xmin=xmin, xmax=xmax, ymin=ymin, ymax=ymax), color="grey20", alpha=0.6, inherit.aes = FALSE)

rect <- data.frame (xmin=31500000, xmax=35800000, ymin = -(width) + alt, ymax = width + alt)

c <- c + geom_rect(data=rect, aes(xmin=xmin, xmax=xmax, ymin=ymin, ymax=ymax), color="grey20", alpha=0, inherit.aes = FALSE)

rect <- data.frame (xmin=35800000, xmax=37800000 ,ymin = -(width) + alt, ymax = width + alt)

c <- c + geom_rect(data=rect, aes(xmin=xmin, xmax=xmax, ymin=ymin, ymax=ymax), color="grey20", alpha=0.5, inherit.aes = FALSE)

rect <- data.frame (xmin=37800000, xmax=39700000, ymin = -(width) + alt, ymax = width + alt)

c <- c + geom_rect(data=rect, aes(xmin=xmin, xmax=xmax, ymin=ymin, ymax=ymax), color="grey20", alpha=0, inherit.aes = FALSE)

rect <- data.frame (xmin=39700000, xmax=42600000, ymin = -(width) + alt, ymax = width + alt)

c <- c + geom_rect(data=rect, aes(xmin=xmin, xmax=xmax, ymin=ymin, ymax=ymax), color="grey20", alpha=0.5, inherit.aes = FALSE)

rect <- data.frame (xmin=42600000, xmax=48129895,ymin = -(width) + alt, ymax = width + alt)

c <- c + geom_rect(data=rect, aes(xmin=xmin, xmax=xmax, ymin=ymin, ymax=ymax), color="grey20", alpha=0, inherit.aes = FALSE)

return(c)

}
